# Person-centered abortion care scale: Validation for medication abortion in the United States^☆^

**DOI:** 10.1016/j.contraception.2024.110485

**Published:** 2024-05-15

**Authors:** May Sudhinaraset, Jessica D. Gipson, Michelle K. Nakphong, Brenda Soun, Patience A. Afulani, Ushma D. Upadhyay, Rajita Patil

**Affiliations:** a Jonathan and Karin Fielding School of Public Health, University of California, Los Angeles, CA, United States; b UCLA Bixby Center to Advance Sexual and Reproductive Health Equity, University of California, Los Angeles, CA, United States; c Division of Prevention Science, Department of Medicine, University of California, San Francisco, CA, United States; d Epidemiology and Biostatistics Department, University of California, San Francisco, CA, United States; e Department of Obstetrics, Gynecology, and Reproductive Sciences, University of California, San Francisco, CA, United States; f David Geffen School of Medicine, Department of Obstetrics and Gynecology, University of California, Los Angeles, CA, United States

**Keywords:** Abortion, Medication abortion, Person-centered care, Quality of care

## Abstract

**Objective:**

Medication abortions now make up the majority of abortions in the US, with new service delivery models such as telehealth; however, it is unclear how this may impact patient experiences. The objective of the study is to adapt and validate a person-centered abortion care (PCAC) scale for medication abortions that was developed in a global South context (Kenya) for use in the United States.

**Study design:**

This study includes medication abortion patients from a hospital-based clinic who had one of two modes of service delivery: (1) telemedicine with no physical exam or ultrasound; or (2) in-person with clinic-based exams and ultrasounds. We conducted a sequential approach to scale development including: (1) defining constructs and item generation; (2) expert reviews; (3) cognitive interviews (*n* = 12); (4) survey development and online survey data collection (*N* = 182, including 45 telemedicine patients and 137 in-person patients); and (5) psychometric analyses.

**Results:**

Exploratory factor analyses identified 29-items for the US-PCAC scale with three subscales: (1) Respect and Dignity (10 items), (2) Responsive and Supportive Care (nine items for the full scale, one additional mode-specific item each for in-person and telemedicine), and (3) Communication and Autonomy (10 items for the full scale, one additional item for telemedicine). The US-PCAC had high content, construct, and criterion validity. It also had high reliability, with a standardized alpha for the full 29-item US-PCAC scale of 0.95. The US-PCAC score was associated with overall satisfaction.

**Conclusion:**

This study found high validity and reliability of a newly-developed person-centered abortion care scale for use in the US. As medication abortion provision expands, this scale can be used in quality improvement efforts.

**Implications:**

This study found high validity and reliability of a newly-developed person-centered care scale for use in the United States for in-person and telemedicine medication abortion.

## 1. Introduction

In June 2022, the US Supreme Court overturned the 1973 Roe v. Wade decision, eliminating federal protections for the provision of abortion. As of February 2024, abortion is illegal in 14 states and at risk of being highly restricted or banned in 26 states total [1]. Amidst these restrictions and COVID-era changes in healthcare delivery, medication abortions now comprise 53% of all facility-based abortions [2]. While the safety and efficacy of medication abortion are well-established, there are no validated tools to assist health care providers in ensuring high quality and person-centered medication abortion care [3].

Quality of care is defined by the Institute of Medicine as care that is safe, effective, person-centered, timely, efficient, and equitable [4]. In particular, person-centered care, or care that is respectful of and responsive to individuals’ preferences, needs, and values [5], is globally recognized as a distinct component of quality abortion care [6]. However, there is a lack of validated measures for abortion quality, particularly person-centered care [7,8]. Abortion care that is not person-centered can take many forms (e.g., discrimination; lack of pain management) with disproportionate impacts on people of color and other marginalized communities [6]. Those of lower socioeconomic status and in highly restrictive legal settings with deeply embedded social stigma are also more likely to experience care that is not person-centered, contributing to health inequities [6,9]. Person-centered care is essential not only as a human right, but is also positively associated with clinical outcomes and adherence to post-abortion guidance [5,6,10].

The US Food and Drug Administration (FDA) approved direct mailing of medication abortion pills to patients through telemedicine options in April 2021, temporarily modifying the in-person dispensing requirement due to the COVID-19 pandemic. This long-standing requirement, which mandated that the first medication abortion pill (mifepristone) be administered in-person under a clinician’s supervision, was removed by the FDA in January 2023 [11]. Telemedicine medication abortion is safe, effective, and acceptable [12–16]. This is in line with the rise in availability and use of telemedicine services across a number of health sectors in recent years, from perinatal mental health [17] to substance use [18] to primary care services [19]. There is a growing body of literature suggesting that telemedicine advances person-centered approaches by increasing patient satisfaction, decreasing barriers to care [20], and meeting peoples’ preferences for how they receive care (e.g. in clinic vs telemedicine) [17]. To our knowledge, only two studies have examined person-centered care for telemedicine medication abortions. The studies found that it increased pregnant peoples’ options, autonomy and access to timely abortion care by removing healthcare barriers related to geographic distance, childcare, or employment [21,22]. However, both studies were qualitative; more empirical, nuanced understanding of different aspects of person-centeredness is needed, including potential limitations of a telemedicine approach. This study aims to adapt and validate a scale to measure person-centered care for in-person and telemedicine medication abortions in California [23].

## 2. Methods

The Person-Centered Abortion Care (PCAC) scale, which was developed and validated in Kenya [23], served as the basis for adaptation and validation in the US context (US-PCAC). We employed a standard sequential approach to scale development described in detail below [24]. This study was conducted at a large urban academic health clinic wherein eligible patients were given the option to have a medication abortion by telemedicine or in person between June 2018–December 2022.

### 2.1. Defining domains and expert reviews

A technical advisory committee (TAC), comprised of 12 experts (i.e., US-based abortion service providers and researchers) reviewed domains of person-centered reproductive healthcare [10] and the original 26-item PCAC scale developed in Kenya [23]. Domains are the major constructs of person-centered care defined by the literature [10]. Additionally, we conducted a literature review on recent measures of abortion experiences. From the original list, the expert reviewers modified, added, and deleted items, ultimately expanding the list to 44 items. In a follow-up TAC meeting and subsequent training with interviewers, TAC and study team members consolidated items from 44 to 37 items to reduce redundancy and omit items considered to be less relevant in a US setting.

### 2.2. Cognitive interviews

In total, 37 items were tested during the cognitive interviews. Input from the cognitive interviews (*n* = 12) included suggestions for slight wording changes (e.g., adding more specificity to questions regarding wait times), consistency of response options across items, verifying that terms were understood and resonated with the experiences of participants across modalities (e.g., “Did you feel seen and heard by the healthcare team?”), and removing items that were duplicative or less relevant according to study participants (e.g. Did you feel like you were physically treated roughly?). In total, seven items were removed that were duplicative or less relevant. Additional changes to items were based on more substantive input from participants; for example, two items were revised to use the term “decisions” versus “decision” to differentiate between the multiple decisions required in the medication abortion process (e.g., where and when they wanted to take the medication, methods for managing pain) versus the larger “decision” to have an abortion or to have a medication versus procedural abortion. Moreover, three items were added based on participant feedback including “Do you feel you were provided with enough information on what to expect regarding pain or discomfort that could arise from the procedure?” “Did you feel that you could confide in the health care team regarding personal or sensitive information?” and “Did you feel that the healthcare team showed that they care about you?”.

### 2.3. Person-centered abortion care survey

The eligibility criteria for the US-PCAC survey were as follows: (1) had either a medication abortion via telemedicine with no exam or ultrasound between April 1, 2020 and December 31, 2022 (referred in text as “telemedicine” patients) or an in-person medication abortion between June 1, 2018 and December 31, 2022 (referred in text as “in-person” patients); (2) 6-weeks or more from completion of abortion to be able to report outcomes (e.g. abortion completion); (3) able to take the online survey in English; and (4) 18 years or older at time of recruitment. The longer timeframe for the in-person sample was to allow for sufficient sample size given the limited number of abortions performed during the study period.

The study team consulted with the clinic’s Clinical and Translational Science Institute (CTSI) biomedical informatics team to obtain lists of eligible participants. Each eligible participant received a recruitment message containing the personal Qualtrics survey link and passcode via email and/or through the hospital’s secure messaging platform. Participants were directed to the informed consent online page. Once they agreed to participate, they were directed to the 20-minute online survey that included questions on demographics, social, and health outcomes, in addition to the PCAC items.

The survey was conducted from December 2021 to March 2023. A total of 970 patients were contacted, with up to three follow-up reminders. The final sample size was 182 participants (147 in-person and 45 telemedicine patients) resulting in a participation rate of 18.8%. A general rule for minimum sample size needed for conducting factor analysis is three participants times the number of items [25]. Our target sample size was 150 participants per modality (for total of 300 participants), but this was not achieved due to the low volume of abortions in the clinic, particularly for the telemedicine sample. Each respondent who completed the survey was given a $20 electronic gift card.

### 2.4. Psychometric analyses

In total, 33 US-PCAC items were included, with two telemedicine specific items and one in-person specific item (see Table 1). We ran a series of factor analyses for the full sample and then separately for in-person and telemedicine participants. Missingness was low across all variables (< 5%) and we therefore used complete case analysis (see Table 1). All analyses were conducted using Stata [26]. Negative items were reverse coded so that negative responses were coded 0 and best responses coded 3 to obtain a uniform scale. We constructed a correlation matrix and examined item-test correlation, item-rest correlation, and alpha to assess reliability. We then conducted exploratory factor analysis. We first assessed a one-factor solution to assess if there was a global measure of US-PCAC. We examined factor loadings of each item, using a cutoff of 0.30 to determine which items to delete or retain. We used oblique rotation because of the naturally occurring correlation between the rotated factors [27].

We used a scree plot, eigenvalues of factors, and conceptual justifications (e.g., examining how each item is understood and theorized in existing literature) to determine the number of factors to retain. We first assessed a scree plot to visually inspect factors with eigenvalues greater than 1.0 [28]. Cronbach’s alpha was used to examine internal consistency for the full and sub-scales, with 0.70 considered to be acceptable reliability [27]. We named sub-domains based on the factors and what is known from existing literature [10,29].

Lastly, we examined criterion validity by assessing bivariate associations between US-PCAC scales and “satisfaction,” a perceived quality of care measure often used to assess quality outcome [30]. Satisfaction was measured by the question, “Overall, how satisfied were you with the entire process?” Response options corresponded to a four-point Likert-type scale (Not at all, Somewhat, Very, or Extremely) and were dichotomized (0 = Not at all/Somewhat satisfied vs 1 = Very/Extremely Satisfied). We used logistic regression to assess bivariate associations. We also conducted sensitivity analyses using a continuous satisfaction score and results did not differ.

All study procedures were reviewed and approved by an Institutional Review Board and informed consent was received by all participants.

## 3. Results

A total of 182 participants completed all PCAC scale items, including 137 in-person participants and 45 telemedicine participants. Demographic characteristics are presented in Table 2.

### 3.1. Exploratory factor analysis (EFA)

Due to low item-correlation and factor loading, we removed “respect support person” (health care team respectful towards support person). For the full sample of participants, we assessed all items excluding the dropped item “respect support person” (29 items). After examining the eigenvalues for each item, we found that items fit better onto a three-factor solution. Oblique rotation indicated three factors and 15 items with a factor loading > 0.3 loaded positively onto one of the three factors: seven items onto Factor 1 corresponding to the Respect and Dignity sub-domain; five items onto Factor 2 corresponding to Responsive and Supportive Care; three items onto Factor 3 corresponding to Communication and Autonomy. Of items that cross-loaded to more than one factor above the cutoff, we categorized four items based on the factor with the higher loading. The remaining items were categorized on conceptual reasoning. Four items loaded highest on Factor 1 but were categorized into other factors for conceptual reasons: “Confidential” (feeling that the health care team kept health information confidential) was categorized into Factor 2; and “Involved,” (provider involved in decisions about care) “Questions,” (could ask health care team any questions) and “Answers” (get answers to all questions in a satisfactory manner) into Factor 3. Three items that loaded highest onto Factor 4 were categorized for conceptual reasons: “Treat negatively” (treated negatively based on identities or characteristics) was grouped into Factor 1, “Overhear” into Factor 2, and “Coerced” (coerced into a decision) into Factor 3. Two items “Support person” and “Language understand” (health care team spoke in understandable language and manner) loaded under the cutoff but was categorized into Factor 1 on a conceptual basis. Appendix 1 presents oblique rotated factor loadings for the 29 items in the final US-PCAC scale and Table 1 summarizes final decisions for each item’s subdomain.

We also conducted factor analyses separately for the in-person and telemedicine samples. For the in-person sample, one additional item “Exams private” (covered up during exams) was added and had a factor loading > 0.3 in Factor 1. For the telemedicine sample, two additional items were administered “Communicate telemedicine” (communicate effectively using telemedicine portal) and “Telemedicine private” (telemedicine visit felt private and secure). “Communicate telemedicine” had a factor loading of 0.4971 in Factor 2 but was categorized under Communication and Autonomy for conceptual reasons. “Telemedicine private” loaded under the cutoff but was retained and categorized under Responsive and Supportive care on conceptual bases.

Table 3 presents standardized alphas and scale descriptive statistics for the full US-PCAC scale and subscales, standardized to a 100-point scale. For the full sample, the standardized alpha for the 29-item PCAC scale was 0.95 (mean score = 87.86, SD = 15.03; Range 20.69–100). For the in-person sample, the standardized alpha for the 30-item PCAC scale was also 0.95 (mean score = 85.94, SD = 16.14; Range 22.22–100). Due to the small sample of telemedicine participants, the standardized alpha was unable to be calculated for the 31-item US-PCAC scale. The unstandardized alpha for the telemedicine 31-item scale was 0.86 (mean score = 94.24, SD = 7.22; Range = 61.29–100).

### 3.2. Criterion validity

In bivariate results, among the full sample, each one-unit increase in total standardized US-PCAC score was associated with a 1.11 times (95% CI: 1.07, 1.15) higher odds of satisfaction (Table 4). All PCAC subscales were also positively associated with satisfaction: Respect and Dignity (OR = 1.06, 95% CI: 1.04, 1.09), Responsive and Supportive Care (OR = 1.06, 95% CI: 1.03, 1.09), and Communication and Autonomy (OR = 1.12, 95% CI: 1.08, 1.17). For the in-person subsample, participants who reported greater satisfaction with the entire process had significantly higher US-PCAC total and subscale scores.

## 4. Discussion

This study is significant in that it is the first validated quality of care scale for abortion in the US and highlights three dimensions of care: respect and dignity, communication and autonomy, and responsive and supportive care. Our study found high construct, content, criterion validity and reliability for the PCAC scale in a US setting for both in-person and telemedicine medication abortion care. Given evidence of improved clinical and patient outcomes associated with patient-centered care [31,32], the US-PCAC provides a much-needed, standardized tool that may aid monitoring and research efforts.

The US-PCAC scale adds to a set of validated person-centered care scales for reproductive health that include scales for abortion [23], family planning [33], prenatal [34], and intrapartum care [35,36]. While there are other scales that measure person-centered contraceptive care (see [37,38]), having a standardized set of measures across the continuum of sexual and reproductive healthcare allows for comparisons across contexts and health services. Across several studies, the communication and autonomy domain consistently has the lowest scores, suggesting that a focus on ensuring comprehension of medical procedures and patient involvement in shared decision-making may be necessary.

This study has several limitations. First, the small sample size, particularly for the telemedicine group, was not sufficiently robust to validate the scale for the telemedicine-specific sample. However, this study provides exploratory evidence of high construct validity and reliability for the overall scale for the telemedicine sample. Second, to recruit sufficient samples, we expanded our eligibility criteria to those who had in-person abortions in 2018 to present; thereby increasing the possibility for challenges in recalling specifics of their care. Third, this study was only offered in English, limiting our sample to English-proficient participants. Lastly, we recognize the limitations of the commonly-used global “satisfaction” measure to assess criterion validity, including that satisfaction is a product of expectations, such that people with low expectations may report higher satisfaction with poor care; moreover, abortion patients oftentimes report high satisfaction because of high stigma associated with abortion [39]. However, given the lack of gold standard, we use satisfaction to measure the outcome of people’s experiences and recognize the need for future studies to examine person-centered care on other abortion outcomes.

The US-PCAC is unique as it includes items specific to either in-person or telemedicine medication abortion. Person-centered care remains critically important given the expansion of telemedicine medication abortion services during COVID and in the post-Dobbs era [7]. The tool will support broader monitoring and research efforts by establishing guidelines for person-centered abortion quality indicators in the US setting. Given differences in quality of abortion care by setting and patient characteristics, the tool can be used to assess health inequities in person-centered abortion care [6]. Future studies may refine and shorten the number of items as performance metrics for quality improvement efforts in clinic settings in order to provide actionable recommendations to healthcare providers and systems.

## Supplementary Material

Appendix A

## Figures and Tables

**Table 1:** Items for person-centered abortion care scale in California for telemedicine and in person administered in the survey (33 items)

Question	Referred to in text as:	Sub-domain

Did you feel seen and heard by your health care team?	Seen and heard	Respect and Dignity
Did you feel your experience and knowledge were valued by the health care team?	Valued	Respect and Dignity
Did the health care team treat you in a friendly manner?	Friendly	Respect and Dignity
Did you feel like you were treated negatively based on any of your identities or characteristics, such as gender, race/ethnicity, age, religion, income, weight, medical conditions, or other identities or characteristics?	Treat negatively	Respect and Dignity
Did you feel that the health care team showed that they care about you?	Cared	Respect and Dignity
Did you feel that you could confide in the health care team regarding personal or sensitive information?	Confide	Respect and Dignity
Did you feel like you were treated with respect by the health care team?	Respect	Respect and Dignity
Did the health care team speak to you in a language or manner that you could understand?	Language understand	Respect and Dignity
Did you feel like you could have a non-medical support person (partner, friend, or family member) with you during your appointment if you wanted anyone there?	Support person	Respect and Dignity
Did you trust your health care team with regards to your medical care?	Trust	Respect and Dignity
Did you feel like the health care provider involved you in decisions about your health care?	Involved	Communication and Autonomy
Did you feel coerced or pressured into a decision by the health care team at any point?	Coerced	Communication and Autonomy
Did the health care team explain the treatment process in a way that you could understand?	Understand treatment	Communication and Autonomy
Did you feel you could ask the health care team any questions you had?	Questions	Communication and Autonomy
Did you get answers to all of your questions in a satisfactory manner?	Answers	Communication and Autonomy
[Telemedicine only] Did you feel you were able to communicate effectively with the health care team using the telemedicine portal?	Communicate telemedicine	Communication and Autonomy
Did you feel like you received enough information to help you make decisions during your care?	Info decisions	Communication and Autonomy
Do you feel you were provided with enough information on what to expect regarding pain or discomfort that could arise from the procedure?	Info expect pain	Communication and Autonomy
Do you feel you were provided with enough information on how to control any pain or discomfort that could arise from the procedure?	Info control pain	Communication and Autonomy
Do you feel you were provided with enough information about if or when to seek emergency care if complications arise?	Info emergency care	Communication and Autonomy
Do you feel that you were given enough resources for help if needed during or after the treatment, such as psychological, social, or medical support?	Help resources	Communication and Autonomy
Did the health care team introduce themselves to you when you first saw them?	Introduce	Responsive and Supportive Care
Did the health care team call you by your preferred name?	Name	Responsive and Supportive Care
Did the health care team use inclusive language, such as your correct pronouns, like she, he, or they?	Inclusive Language	Responsive and Supportive Care
How do you feel about the amount of time it took to get your first appointment?	Time to appointment	Responsive and Supportive Care
How do you feel about the amount of time you spent waiting to be seen by your health care provider during your visit(s)?	Time waiting	Responsive and Supportive Care
How do you feel about the amount of time from your initial appointment to when you received your medication?	Time to medication	Responsive and Supportive Care
How do you feel about the amount of time the health care provider spent with you?	Time with provider	Responsive and Supportive Care
When you were speaking to the health care team, did you feel that other people could overhear what you were discussing?	Overhear	Responsive and Supportive Care
Do you feel that your health care team keeps your health information confidential?	Confidential	Responsive and Supportive Care
[Telemedicine only] Did your telemedicine visit(s) feel private and secure?	Telemedicine private	Responsive and Supportive Care
[In-person only] During exams, were you covered up so that you did not feel exposed?	Exams private	Responsive and Supportive Care
*Was the healthcare team respectful towards your support person(s)?*	*Respect support person*	*Deleted*

The “Respect support person” item, marked in italics, was dropped from the final scale.

**Table 2:** Demographic characteristics, by in-person and telemedicine clients in California, 2021–2023

Characteristic	Total (*n* = 182) *N* (%)	In-person (*n* = 137) *N* (%)	Telemedicine (*n* = 45) *N* (%)	*p*-value

Age, years	Mean = 30.34 (SD 6.71)	Mean = 30.74 (SD 6.89)	Mean = 29.11 (SD 6.03)	0.121
24 and under	42 (23.1%)	30 (21.9%)	12 (26.7%)	
25–29	47 (25.8%)	33 (24.1%)	14 (31.1%)	
30–34	42 (23.1%)	30 (21.9%)	12 (26.7%)	
35+	47 (25.8%)	40 (29.2%)	7 (15.6%)	
Missing	4 (2.2%)	4 (2.9%)	0 (0%)	
Gender				0.726
Female	178 (97.8%)	133 (97.1%)	45 (100%)	
Male	1 (0.5%)	1 (0.7%)	0 (0.0%)	
Non-binary/genderqueer/gender non-conforming	2 (1.1%)	2 (1.5%)	0 (0.0%)	
Other	1 (0.5%)	1 (0.7%)	0 (0.0%)	
Relationship status				0.847
Divorced	5 (2.7%)	3 (2.2%)	2 (4.4%)	
Living with partner	31 (17.0%)	22 (16.1%)	9 (20.0%)	
Married	47 (25.8%)	36 (26.3%)	11 (24.4%)	
Never married	96 (52.7%)	73 (53.3%)	23 (51.1%)	
Missing	3 (1.6%)	3 (2.2%)	0 (0.0%)	
Race (select all that apply)^a^				
American Indian or Alaskan Native	7 (3.9%)	6 (4.4%)	1 (2.2%)	0.514
Asian or Asian American	35 (19.2%)	30 (21.9%)	5 (11.1%)	0.110
Black or African American	25 (13.7%)	18 (13.1%)	7 (15.6%)	0.682
North African or Middle Eastern	5 (2.8%)	2 (1.5%)	2 (6.7%)	0.064
White	78 (42.9%)	56 (40.9%)	22 (48.9%)	0.437
None of the above or mixed	39 (21.4%)	30 (21.9%)	9 (20.0%)	0.788
Missing	6 (3.3%)	5 (3.7%)	1 (2.2%)	
Latino/a or Hispanic				0.783
No	115 (63.2%)	87 (63.5%)	28 (62.2%)	
Yes	66 (36.3%)	49 (35.8%)	17 (37.8%)	
Missing	1 (0.5%)	1 (0.7%)	0 0.0%)	
Education				0.408
High school graduate, GED, or equivalent	7 (3.8%)	4 (2.9%)	3 (6.7%)	
Some college, junior college, or vocational	43 (23.6%)	32 (23.4%)	11 (24.4%)	
College graduate (BA, BS)	84 (46.2%)	67 (48.9%)	17 (37.8%)	
Graduate or professional degree	48 (26.4%)	34 (24.8%)	14 (31.1%)	
Employment				0.373
Not currently employed	26 (14.3%)	20 (14.6%)	6 (13.3%)	
Employed, full-time	120 (65.9%)	93 (67.9%)	27 (60.0%)	
Employed, part-time	36 (19.8%)	24 (17.5%)	12 (26.7%)	
Number of pregnancies (including most recent)	Mean = 2.01 (SD 1.33)	Mean = 2.10 (SD 1.40)	Mean = 1.73 (SD 1.05)	0.107
1	91 (50.0%)	64 (46.7%)	27 (60.0%)	
2	34 (18.7%)	26 (19.0%)	8 (17.8%)	
3	31 (17.0%)	26 (19.0%)	5 (11.1%)	
4	15 (8.2%)	10 (7.3%)	5 (11.1%)	
5 or more	9 (4.9%)	9 (6.6%)	0 (0.0%)	
Missing	2 (1.1%)	2 (1.5%)	0 (0.0%)	
Number of abortions (including most recent)	Mean = 1.34 (SD 0.71)	Mean = 1.39 (SD 0.76)	Mean = 1.2 (SD 0.50)	0.121
1	134 (73.6%)	96 (70.1%)	38 (84.4%)	
2	33 (18.1%)	28 (20.4%)	5 (11.1%)	
3 or more	11 (6.0%)	9 (6.6%)	2 (4.4%)	
Missing	4 (2.2%)	4 (2.9%)	0 (0.0%)	
Number of living children	Mean = 0.51 (SD 0.82)	Mean = 0.53 (SD 0.84)	Mean = 0.43 (SD 0.76)	0.495
0	121 (66.5%)	89 (65.0%)	32 (71.1%)	
1	28 (15.4%)	23 (16.8%)	5 (11.1%)	
2	26 (14.3%)	19 (13.9%)	7 (15.6%)	
3	4 (2.2%)	4 (2.9%)	0 (0.0%)	
Missing	3 (1.6%)	2 (1.5%)	1 (2.2%)	
Health Insurance and type				0.039
Private or employer-provided insurance	142 (78.0%)	102 (74.5%)	40 (88.9%)	
Medicaid/Medical/Medicare/Tricare/Govt insurance	24 (13.2%)	22 (16.1%)	2 (4.4%)	
No insurance	7 (3.8%)	5 (3.6%)	2 (4.4%)	
Other	8 (4.4%)	8 (5.8%)	0 (0.0%)	
Missing	1 (0.5%)	0 (0.0%)	1 (2.2%)	

BA, bachelor of arts; BS, bachelor of science; GED, General education development.

a Percentages for race total greater than 100% because participants could select more than one racial category.

**Table 3:** Standardized alphas and means for PCAC scale and sub-domains stratified by In-person and Telemedicine clients in California, 2021–2023

	Alpha (standardized)	Mean Score	SD	Min	Max

*Full Sample*					
Full PCAC Scale (29 items)	0.95	87.86	15.03	20.69	100
Respect and Dignity (10 items)	0.91	88.32	17.41	13.33	100
Responsive and Supportive Care (nine items)	0.84	87.77	15.46	14.81	100
Communication and Autonomy (10 items)	0.90	87.49	17.23	13.33	100
*In-person*					
Full PCAC Scale (30 items)	0.95	85.94	16.14	22.22	100
Respect and Dignity (10 items)	0.91	86.06	18.95	13.33	100
Responsive and Supportive Care (10 items)	0.84	86.06	15.98	20	100
Communication and Autonomy (10 items)	0.91	85.69	18.70	13.33	100
*Telemedicine* Full PCAC Scale (31 items)	0.86^a^	94.24	7.22	61.29	100
Respect and Dignity (10 items)	0.87	95.19	8.54	53.33	100
Responsive and Supportive Care (10 items)	0.67^a^	94.59	7.70	60	100
Communication and Autonomy (11 items)	0.82	93.06	10.00	60.61	100

PCAC, person-centered abortion care.

a Unstandardized alpha. Unable to calculate standardized alpha.

**Table 4:** Bivariate logistic regressions of person-centered abortion care standardized full and sub-scales and quality of care measures, 2021–2023

	Perceived quality of care measures
	Satisfaction
	OR (95% CI)

*Full Sample*	
Full Person-Centered Abortion Care Scale (29 items)	1.11^a^
	(1.07–1.15)
Respect and Dignity (10 items)	1.06^a^
	(1.04–1.09)
Responsive and Supportive Care (nine items)	1.06^a^
	(1.03–1.09)
Communication and Autonomy (10 items)	1.12^a^
	(1.08–1.17)
*In-person* Full Person-Centered Abortion Care Scale (30 items)	1.14^a^ (1.08–1.20)
Respect and Dignity (10 items)	1.08^a^
	(1.05–1.11)
Responsive and Supportive Care (10 items)	1.08^a^
	(1.05–1.12)
Communication and Autonomy (10 items)	1.16^a^
	(1.10–1.22)
*Telemedicine*	
Full Person-Centered Abortion Care Scale (31 items)	1.06
	(0.96–1.16)
Respect and Dignity (10 items)	1.02
	(0.94–1.10)
Responsive and Supportive Care (10 items)	1.00
	(0.90–1.11)
Communication and Autonomy (11 items)	1.07^b^
	(1.00–1.15)

a *p* < 0.001.

b *p* < 0.05.
