# Supplementary material for: Person-centered abortion care scale: Validation for medication abortion in the United States
Source: Contraception. Author manuscript; Available in PMC 2025 Feb 24. (PMC11849315; doi:10.1016/j.contraception.2024.110485)
Supplement: Appendix A [file NIHMS2030580-supplement-Appendix_A.docx]

**Appendix**

**Appendix 1. Rotated factor loadings for overall sample, overall PCAC score and sub-domain scores**

|  | Rotated Factor Loadings | | | |
| --- | --- | --- | --- | --- |
| Item | Full PCAC scale (29 items) | Sub-scales | | |
|  |  | Respect & Dignity (10 items) | Responsive & Supportive Care (9 items) | Communication & Autonomy (10 items) |
| Seen & heard | 0.8391 | 0.8391 |  |  |
| Valued | 0.8700 | 0.8899 |  |  |
| Friendly | 0.7915 | 0.8644 |  |  |
| Treat negatively | 0.4023 | 0.4088 |  |  |
| Cared | 0.8375 | 0.8667 |  |  |
| Confide | 0.8063 | 0.8567 |  |  |
| Respect | 0.8595 | 0.9397 |  |  |
| Language understand | 0.3613 | 0.3944 |  |  |
| Support person | 0.2889 | 0.3194 |  |  |
| Trust | 0.7875 | 0.8101 |  |  |
| Time to appointment | 0.4601 |  | 0.6008 |  |
| Time waiting | 0.5594 |  | 0.6762 |  |
| Time to medication | 0.4567 |  | 0.5828 |  |
| Time with provider | 0.6956 |  | 0.6446 |  |
| Introduce | 0.5241 |  | 0.6115 |  |
| Name | 0.5989 |  | 0.6667 |  |
| Inclusive language | 0.4678 |  | 0.5618 |  |
| Overhear | 0.2973 |  | 0.3742 |  |
| Confidential | 0.6779 |  | 0.6509 |  |
| Involved | 0.5454 |  |  | 0.5216 |
| Coerced | 0.4694 |  |  | 0.3607 |
| Understand treatment | 0.7188 |  |  | 0.7908 |
| Questions | 0.7890 |  |  | 0.7752 |
| Answers | 0.8331 |  |  | 0.8133 |
| Info decisions | 0.7798 |  |  | 0.7986 |
| Info expect pain | 0.6637 |  |  | 0.8277 |
| Info control pain | 0.5788 |  |  | 0.7384 |
| Info emergency care | 0.6619 |  |  | 0.7480 |
| Help resources | 0.5312 |  |  | 0.5966 |
